# Thrown in at the deep end? Perceptions of burdens, gains, and contributions to the integration of refugee students among teachers with(out) target group-specific professional knowledge

**DOI:** 10.3389/fsoc.2022.840176

**Published:** 2022-08-16

**Authors:** Franziska Spanner, Elisabeth Maué

**Affiliations:** ^1^Department of Sociology, University of Konstanz, Konstanz, Germany; ^2^Department of Economics, University of Konstanz, Konstanz, Germany

**Keywords:** refugees, preparation classes for refugees, integration, teachers' professional knowledge, professional training, teachers' stress, teachers' success, mixed-methods study

## Abstract

In Germany, young migrants and refugees whose German language skills are not sufficient for attending regular classes at school are assigned to so-called “preparation classes”. As the name implies, the main objective of these classes is to teach German language skills and thereby prepare students for regular classes or vocational education and training. Teachers in these classes face special challenges: They have to teach a group of students that is highly heterogeneous in several key respects, they have to deal with a dearth of institutional support and guidance, and they have a lot of responsibility regarding their students' integration in Germany. Therefore, this paper asks what target group-specific professional knowledge teachers possess and to what extent this knowledge reduces the teachers' burdens and raises their perception of gains and contributions to their students' success. Our mixed-method survey of 48 teachers of refugees in prevocational preparation classes in Germany shows that the majority of teachers acquired useful knowledge for teaching refugees, e.g., dealing with trauma and post-traumatic stress disorder, building German proficiency, or language-sensitive teaching, by attending professional training. In sum, most of the teachers saw immigration to Germany as both a problem and an opportunity. The main burdens they identify are bad working conditions, a lack of support and appreciation and a high demand for high flexibility. Despite generally low stress levels and high resilience, the teachers were extremely stressed by the heterogeneity in their students' performance, especially those teachers who had completed professional training. However, teachers appreciated the students' willingness to learn and gratitude, and perceived cultural diversity in the classroom as a gain for themselves and their teaching. The teachers felt they had mostly contributed to their students' acquisition of the German language as well as their educational progress in general and their personal development. Students' chances of success are expected to be especially high in the German language acquisition and the management of everyday life in Germany. The assessments of teachers with and without target group-specific professional knowledge differ only slightly and rarely significantly.

## Introduction

In the course of immigration from crisis regions, particularly in 2015 and 2016, a large number of children and adolescents came to Germany (Brücker et al., 2017). These children and young people are, like German nationals, subject to compulsory education in Germany and are granted special access to vocational education and training compared to refugees in other countries (Jørgensen et al., 2021). If the integration of refugees is perceived as both the joint of various institutions in the country of immigration (cf. Stöbe-Blossey et al., 2019) and as a reciprocal process between immigrants and natives (Alba and Nee, 2003), the right to education of asylum seekers goes hand in hand with an obligation of educational institutions in Germany to provide the necessary capacity and quality. Hence, educational institutions must both create the basis for the integration efforts of refugees and contribute to integration on the part of the host society (cf. Reinke and Goller, 2022). For immigrant children and young people whose German language skills are not sufficient to attend a regular class, so-called preparation classes have therefore been set up at general education and vocational schools. These preparation classes mainly serve to teach the German language and are intended to facilitate transition to regular courses of education or vocational education and training (Baethge and Seeber, 2016; Emmerich et al., 2017).

Teachers in preparation classes for refugees are representatives of the host society and act as a link between that society and refugees. They are primarily in daily personal contact with the refugees, and responsible for implementing society's expectations of educational institutions, and for advancing the integration goal set by policymakers (Blossfeld et al., 2016). In this context, one of their core tasks is “to offer young people support and confidence after and during a time of disorientation” (Streinz, 2015, p. 6; translation by the authors) as well as to provide them with opportunities not only for linguistic and professional development, but also especially for personal development (ibid.). Accordingly, refugees face the demanding task of acquiring the German language, including obtaining specific qualifications, while also integrating into the education system, the vocational education and training system, the labor market, and society more widely. The responsible institutions, and above all the teachers, also face major challenges. For integration to succeed, appropriate resources must be made available to enable the successful accomplishment of this task alongside meeting associated challenges.

However, recent public and academic discourse often assumes a one-sided understanding of integration (Glorius and Schondelmayer, 2019, p. 222) and is therefore largely focused on the expectations of refugees (cf. Matzner, 2012, p. 12). Thus, many studies focus on refugees themselves as the target group to be educated (e.g., Calmbach and Edwards, 2019; Maué et al., 2021). In contrast, there has been little research on the teachers of refugees in terms of the challenges the teachers face or regarding positive aspects they relate to their job (e.g., for Germany: Karakayali et al., 2017; for Lebanon: Khansa and Bahous, 2021; Weiß et al., 2021; for Sweden: Asghari, 2022; for the U.S.: Damaschke-Deitrick et al., 2022; Reinke and Goller, 2022). Yet, it is the teachers who support refugees in their education and their progress toward an independent life in the host society. To do so, the teachers require a range of skills including how to deal with trauma and specific teaching materials for the respective target group (ibid.). Previous research on teachers in preparation classes for refugees has mainly been based exclusively on qualitative research or case studies conducted in individual German federal states (e.g., Scheiermann and Walter, 2016; Kärner et al., 2019; Stöbe-Blossey et al., 2019; Weiß et al., 2021).

Against this background, this article explores how teachers in preparation classes experience their task of contributing to the integration of young refugees, a process that affects the education system as well as society as a whole. We use an interdisciplinary and mixed methods approach (Tashakkori and Creswell, 2007): First, teachers at vocational schools in southwestern Germany are examined from a perspective of migration sociology and educational science. Second, we analyze answers to open questions qualitatively, and answers to standardized closed questions quantitatively. The results from these analyses are combined to provide a comprehensive description of teachers' perceived burdens and gains from teaching refugee students, as well as of the contributions of the teachers to their students' integration. Furthermore, we investigate the correlation of teachers' burdens, gains, and contributions to students' integration with the teachers' target group-specific professional knowledge, including teachers' attitudes toward immigration. This knowledge can be gained by means of specific professional training,^1^ for example on aspects of acquiring the German language or dealing with traumatized students.

The first part of this article clarifies the understanding about refugee integration and the importance of (school) educational processes in general and teachers in particular (Section The special role of teachers for the integration of refugees). Subsequently, the focus is on teachers' potential burdens when teaching refugees (Section Potentially stressful conditions when teaching refugees) and their target group-specific professional knowledge (including attitudes) that is needed to deal with such burdens and to provide good target-group oriented lessons for refugees (Section Target group-specific professional knowledge of teachers). The description of the context of schooling for refugees in Germany and in Baden-Württemberg, where the study was conducted, is provided in Section The case of Baden-Württemberg in the German context. Methodological explanations (Section Data and methods) are followed by the presentation of central findings (Section Results) and their discussion (Section Discussion).

## The special role of teachers for the integration of refugees

According to human capital theory, education should be seen as an investment in the integration of immigrants (cf. Chiswick, 1978; Chiswick and Miller, 1996). Refugees sometimes either have received little education from their country of origin (Diehl et al., 2017; Brenzel and Kosyakova, 2018) or often cannot ‘transfer' their education to the host country (Chiswick, 1978; Friedberg, 2000; Hunkler et al., 2021; Jørgensen et al., 2021). However, since education is both indirectly and directly formative for life chances in the host society, for example regarding the labor market (Diehl et al., 2016a, p. 4), many refugees want to, and in many cases must, (re)acquire specific resources in the host country (Liebau and Salikutluk, 2016).

Teachers are actively involved in making refugees' individual learning processes successful by supporting the acquisition of certain forms of human capital, especially language skills and educational certificates that are closely related to the immigrants' opportunities in the labor market (cf. Kalter, 2005; Reinke and Goller, 2022). Education – especially vocational education and training – can thus be considered as a “key to integration” (Stöbe-Blossey et al., 2019, p. 295; also Jørgensen et al., 2021).

We understand the term integration as “the incorporation of the individual members of ethnic groups into the various spheres of the host society” (Esser, 2000, p. 286; translation by the authors) – so-called social integration. Education, and especially teachers, play a crucial role in all four dimensions of social integration.

1. *Culturation* describes the acquisition of certain knowledge and skills that enable actors to interact and take action (Esser, 2000, p. 272). In the context of immigrant integration, the main focus of culturation is often on the acquisition of the language of the host country (cf. Esser, 2006, p. 26), for which schooling is particularly significant (Chiswick and Miller, 1995, 1996; Becker-Mrotzek et al., 2021). Moreover, language acquisition again critically influences learning in other subjects (Schipolowski et al., 2021). Teachers can contribute to improving the language skills of refugees both in regular language classes (cf. Schründer-Lenzen, 2012) and in classes specifically designed for migrants learning a second language (cf. Rösch, 2012), which often takes place in specific preparation classes. In addition, there are opportunities and needs for language-sensitive teaching in other subjects, not only to consider the different language levels of students, but also to actively promote the acquisition of the German language both in terms of educational and technical language (Bickes and Steuber, 2017; Roche and Terrasi-Haufe, 2019; Becker-Mrotzek et al., 2021). Gogolin et al. (2020) have developed quality criteria for the teaching of continuous language education in linguistically heterogeneous classes that teachers can use as a guideline. These include, for example, individual language level diagnostics and the provision of general and educational language resources. Furthermore, teachers are in contact with their students outside the classroom so that refugees can additionally practice their communication in the language of the host country in such interactions (cf. Kniffka and Siebert-Ott, 2009).
2. *Placement* is the occupation of positions by actors in socially relevant areas, such as the labor and housing market or the education system (Esser, 2006, p. 26). To achieve a good position in the labor market, for example, one needs to meet the relevant prerequisites, such as educational qualifications^2^ and additional qualifications (e.g., Heath and Cheung, 2007; Hunkler et al., 2021; Jørgensen et al., 2021). Teachers play a crucial role in the attainment of educational qualifications because their teaching provides the subject-specific and cross-curricular foundations for successful completion (Reinke and Goller, 2022). Language teaching is an essential element in this regard, as knowledge of the language of instruction is closely related to academic achievement (Kempert et al., 2016, p. 158; Schipolowski et al., 2021).
3. *Interaction* refers to making social contacts (Esser, 2006, p. 26). School as a place of encounter can provide the resources useful for building networks. School provides opportunities for interethnic contacts that serve to reduce prejudices on the part of both the majority society and the immigrants (Allport, 1979, p. 281) and can have a positive impact on the education of refugees (e.g., Hanushek et al., 2009; van Ewijk and Sleegers, 2010) and their integration into the labor market (e.g., Kalter, 2006; Lancee and Hartung, 2012; Kalter and Kogan, 2014; Jørgensen et al., 2021). It is up to teachers to create a classroom climate that facilitates interethnic contact by reducing perceived cultural distance and creating positive norms of contact (Thijs and Verkuyten, 2014; Schachner et al., 2015).
4. *Identification* occurs when individual actors identify with the social system and feel they belong to a society (Esser, 2006, p. 26). At schools, as institutions for the transmission of social values and norms (Karakaşoglu, 2013, p. 127), teachers can both stimulate development toward a national identity in the classroom and, for instance in social studies classes, promote the adoption of values of the host society (cf. Hardwick et al., 2010). In addition, teachers can raise awareness of different cultures, e.g., during (foreign) language lessons (Yuan and Fang, 2016), and emphasize the value of multilingualism as a resource (Gutentag et al., 2018; Becker-Mrotzek et al., 2021).

These four dimensions of social integration are not independent of each other but are interconnected or mutually dependent. To give one example, certificates and competencies acquired in educational institutions are not only beneficial for professional success, but also for participation in other areas of society, such as contacts with natives (Diehl et al., 2016b). They increase the chances of establishing contacts with locals and can thus promote linguistic (cultural) integration as well as identification (Diehl et al., 2016a, p. 4). Yet, the role of teachers – who additionally act as advisors when teaching refugees (Sari and Kenner, 2017; Simml, 2019) – as well as their work contents differ in essential ways from teaching natives or regular classes, e.g., including the need to focus on German language acquisition or the consideration of the special life circumstances of the students (Simml, 2019; Reinke and Goller, 2022). Therefore, the key role that education has in the integration of migrants means that teachers of refugees have to target a wide range of objectives. This is not only potentially stressful but also points to the importance of target group-specific professional knowledge. Furthermore, teachers' contributions to their students' integration may also be oriented toward these different and various teaching goals.

## Potentially stressful conditions when teaching refugees

Teaching is a particularly challenging and stressful profession (Rudow, 2000; Rothland, 2013). The reasons for this include problems with student discipline, administrative tasks, heterogeneous class compositions, or unclear work requirements (van Dick, 1999). The stress teachers feel in their job can have negative consequences for themselves, for example increasing motivation to leave the profession (e.g., Skaalvik and Skaalvik, 2016) or the risk of burnout (Gutentag et al., 2018). Teacher stress can also have negative consequences for their students' education, potentially resulting, for instance, in lower learning achievement (e.g., Klusmann et al., 2016).

With regard to teaching in preparation classes for refugee students, there are additional stress factors. This is due to the special circumstances of refugee students in contrast to “regular” migrants and native students. Experiences in countries of origin, the majority of which are characterized by war and violence, (traumatic) experiences associated with fleeing, and an uncertain residence status can be psychosocially stressful and even cause post-traumatic stress disorder in students (Kärner et al., 2016; Weiß et al., 2021), sometimes preventing them from attending class or indeed learning altogether (cf. Boketta and Sachser, 2012; Massumi, 2019; Weiß et al., 2021; Reinke and Goller, 2022). Teachers of refugees often have difficulties dealing with the trauma of their students (Kärner et al., 2016) or lack the appropriate skills to deal with traumatized students (Weiß et al., 2021; Damaschke-Deitrick et al., 2022). This can ultimately lead to feelings of individual failure or being left alone among teachers (Müller, 2021; Reinke and Goller, 2022). Furthermore, refugees are less self-selective in terms of characteristics for successful integration into the host country than are other migrant groups. Therefore, comparatively less investment and preparation for migration can be expected (Chiswick and Miller, 1995, p. 251). Despite individual and country of origin differences in educational and vocational experiences, refugee youth in Germany overall have lower average levels of formal education (Schier, 2017; Stoewe, 2017), which is further reduced by longer interruptions in their educational trajectories (Diehl et al., 2017; Schier, 2017; Hunkler et al., 2021). Moreover, their prerequisites for acquiring the German language differ from those of “regular” migrants (Massumi, 2019). Teachers of refugees thus have various tasks that may go beyond the knowledge they acquired during teacher education. These tasks for example entail teaching the students how to learn (again), compensating students' knowledge gaps that resulted from earlier interruptions in their educational career, and considering students' worries about their families and friends left behind or about their refugee status.

Nonetheless, it should be emphasized that refugee students do not represent a homogeneous group. On the contrary, in preparation classes for refugee children and youths, there is considerable heterogeneity of the students in terms of educational background, language skills, performance, motivation, and cultural and religious background (Baumann and Riedl, 2016; Diehl et al., 2017; Maué et al., 2018, 2021; Reinke and Goller, 2022). Heterogeneity is most often referred to as “differences between individuals based on so-called sociocultural categories of difference” (Waldis et al., 2021, p. 202) and, in the school context, also “learning and performance-related differences” (ibid.). Teachers need to account for heterogeneity in different ways depending on its expression in different dimensions (Kärner et al., 2016; Massumi, 2019). For example, teaching materials must be provided for different proficiency levels (Glorius and Schondelmayer, 2019) and different teaching methods must be used to accommodate heterogeneous class settings (Weinert, 1997). Intercultural competence (Gruber and Fiebig, 2013; Terhart and von Dewitz, 2018; Ehmann, 2021) and language-sensitive teaching (Bickes and Steuber, 2017; Roche and Terrasi-Haufe, 2019; Becker-Mrotzek et al., 2021) can also be beneficial for successful teaching in preparation classes. However, this requires specific knowledge on the part of the teachers (Gibbons, 2015) and is accompanied by an increased effort on their part in lesson planning and lesson design.

In addition to the particularities of the students, teachers in preparation classes face structural challenges. First, teachers are under great pressure to support students in transitioning successfully to regular classes and to close prior knowledge gaps (Schipolowski et al., 2021) in order to increase the students' chances of completing their regular classes, graduating from high school, and succeeding in integrating in general. Second, in particular shortly after implementation of the preparation classes, when the teachers of our study were surveyed, teachers had to cope with fuzzy guidelines instead of binding curricula (Karakayali et al., 2017) that teachers of regular classes have. Also, the criteria and regulations regarding students' transition from preparation to regular classes were unclear (Karakayali et al., 2017). Finally, and also in contrast to “regular” teachers, teachers of refugee youths have limited resources such as (target group-appropriate) working materials, especially with regards to the acquisition of the German language (Reinke and Goller, 2022). In a survey of teachers of refugees at middle schools and vocational schools in Bavaria (Kärner et al., 2019), 12 out of 16 teachers cited organizational conditions, such as the lack of appropriate teaching and learning materials, as limits to the use of internal differentiation measures^3^. This discrepancy between aim and means aggravated even more by the short time frame that teachers have to achieve the aim. In the case of prevocational preparation classes, where the number of class repetitions is limited, this time frame is a maximum of 2 or 3 years.

Likewise, the often precarious employment conditions of teachers in preparation classes should be noted as a potential stress factor (Karakayali et al., 2017): In contrast to regular teachers, preparation class teachers often do not hold a civil servant status and instead hold short-term employment contracts that do not include summer vacations. Moreover, newly hired staff that have changed career often receive lower earnings than regular teachers.

The difficult structural conditions, and the special composition of the students, make educating refugees – despite their high aspirations (Brücker et al., 2017; Maué et al., 2018; Schneider, 2018) and self-efficacy (Diehl et al., 2017) – a challenging task. It should not be overlooked that schools and teachers bear the “main burden of educational integration” (Blossfeld et al., 2016, p. 16). Given this fact, it is also important to consider in the analyses the gains that motivate teachers in this job and that might help them to cope with the difficulties of the job (cf. Simml, 2019). Including positive aspects of the job in our analyses thus adds to existing research, which has so far been primarily focused on the stresses and strains of teachers.

## Target group-specific professional knowledge of teachers

What knowledge, skills, and attitudes do teachers need to support the education and integration of refugee students, while also coping with particular stresses that arise from e.g., heterogeneous class composition or unusual behaviors in the class (Kärner et al., 2016; Karakayali et al., 2017; Reinke and Goller, 2022)? A qualitative SINUS study by Calmbach and Edwards (2019) shows that young refugees judge their teachers by their “sensitivity or [...] demand responsiveness with regard to the specific situation of young refugees, good lesson preparation, openness to or patience with queries, and discipline or authority” (p. 81; translation by the authors). The lack of language sensitivity of teachers can contribute to students' dissatisfaction with instruction to a large extent (Calmbach and Edwards, 2019). Kärner et al. (2019) show that teachers can fulfill this challenging and demanding task. They identify internal differentiation measures as an important method by which teachers can address the individual needs of young refugees and foster a successful integration process.

To meet the particular requirements of teaching refugees, teachers need specific knowledge adapted to the target group. Baumert and Kunter (2011), p. 33; refer to the knowledge and skills of teachers in terms of professional knowledge as the “core of professionalism.” Professional knowledge is concretized as subject knowledge, subject didactic knowledge, pedagogical-psychological knowledge, and organizational and counseling knowledge. In addition, self-regulatory skills, motivational orientations, and convictions and values all play a significant role (ibid.). In the following paragraphs, different forms of target group-specific professional knowledge relevant to teachers in preparation classes are outlined.

Teachers in preparation classes for refugees need an understanding of the heterogeneity of the students. This is part of pedagogical-psychological knowledge and thus part of professional knowledge (Voss and Kunter, 2013). The pedagogical-psychological knowledge refers to knowledge about student learning processes as well as knowledge about classroom and instructional challenges associated with individual student characteristics (knowledge about effective classroom management and knowledge about methods). This knowledge, in turn, can be helpful in developing measures for internal differentiation (cf. Zoyke, 2016). Culturally heterogeneous classes require culturally sensitive classroom management that takes into account multiple dimensions (classroom climate, learning contexts, performance assessment and teaching methods as well as curriculum content). Culturally sensitive classroom management is characterized by appreciation of diversity and respect for unfamiliar cultural backgrounds (Strasser, 2021), fosters cultural inclusiveness, and aims to provide equitable opportunities for all students (Abacioglu et al., 2022).

In addition, multicultural beliefs are important for teaching students with a migration background and dealing with cultural heterogeneity: “teachers with multicultural beliefs can be expected to incorporate students' different cultures into everyday school practice when planning their lessons, choosing materials, and interacting with students in class” (Hachfeld et al., 2011, p. 987). Multicultural beliefs can reduce prejudice and promote self-efficacy, enthusiasm, self-reflection, and culturally responsive teaching (Hachfeld et al., 2011, 2012; Civitillo et al., 2019; Gebauer and McElvany, 2020; Hachfeld and Syring, 2020; Abacioglu et al., 2022), which in turn can reduce strain and burnout (Gutentag et al., 2018; Strasser, 2021). There is little evidence regarding teachers' attitudes toward refugees (e.g., Terzi et al., 2019; Chwastek et al., 2021). However, since hardly any curricula and teaching materials were available, especially at the beginning of the established preparation classes (Reinke and Goller, 2022), and the employment conditions were poor in some cases (Karakayali et al., 2017), it can be assumed that most of the teachers who “take on” this activity have a fundamentally positive attitude toward refugees. Furthermore, many school leaders first asked for teachers to voluntarily take over preparation classes. These voluntary teachers can also be expected to be positively selected regarding their attitudes toward refugee students.

Moreover, migration-related professional knowledge, for example on dealing with trauma and post-traumatic stress disorders (Blossfeld et al., 2016.; Weiß et al., 2021; Damaschke-Deitrick et al., 2022), on the acquisition of the German language, on language-sensitive teaching (Bickes and Steuber, 2017; Roche and Terrasi-Haufe, 2019; Becker-Mrotzek et al., 2021), and on the creation of appropriate teaching materials is required for the target group of refugee students (Boketta and Sachser, 2012; MKJS, 2016). Because such topics are not included in teacher training courses in sufficient detail, additional training is necessary, which requires additional time (and money) investments of teachers.

Furthermore, attachment figures are of high importance for the students in developing positive future perspectives (Sari and Kenner, 2017). In the case of refugees, these are often teachers who are asked for advice on school and extracurricular concerns (Calmbach and Edwards, 2019; Glorius and Schondelmayer, 2019). Thus, those staff need appropriate counseling knowledge (Baumert and Kunter, 2011; Strasser, 2021) and content-related knowledge, for example about adequate consulting services for residence law issues.

Such topics require target group-specific professional training^4^ which generally addresses the “change of professional convictions, subjective theories, and declarative knowledge components of teachers” and “the expansion and flexibilization of instructional action” (Lipowsky, 2011, p. 399; translation by the authors). In addition to a targeted reflection on teaching action, different professional training formats also enable exchange with colleagues (Terhart et al., 2017, p. 241f.). It should be noted, however, that professional training for teachers of refugees is not always offered in sufficient breadth and for enough topics (cf. Scheiermann and Walter, 2016).

The successfulness and effectiveness of professional training for teachers depends on various components. To give one example, continuing education is considered effective if it expands subject didactic knowledge and beliefs (for an overview: Lipowsky, 2011) and thus contributes to higher teaching quality (Lipowsky, 2006). In addition, the perceived relevance of, and motivation(s) for, professional training both play a role in teaching effectiveness. For instance, teachers who attend professional training to expand their knowledge experience less stress in their daily school life (Rzejak et al., 2014). Against this backdrop and given the unfavorable structural framework of teaching (see Section Potentially stressful conditions when teaching refugees), teachers' target group-specific professional knowledge regarding students' particular circumstances could be beneficial in two ways: First, it could reduce perceived stresses caused by the students' special requirements and more difficult teaching conditions. Second, it could make it easier to achieve the goals specified for teaching refugees, such as acquiring the German language or helping refugees find their way in everyday life in Germany. Appropriate professional training is essential for the acquisition of target group-specific professional knowledge and for its adequate use in teaching and counseling.

Ideally, teachers receive the necessary knowledge during their teacher training. However, in Germany's federal system, the federal states have sovereignty over education, both in terms of the design of the education system and the training of teachers. Although there are common standards for teacher education (KMK, 2019), the competencies formulated there are very general and have to be translated into the curricula of the different teacher training programs at the individual universities or universities of education. Accordingly, the curricula differ between the institutions, the teaching profession for different school types, and the school subject, – both within the federal states and within Germany. This also applies to the curricular embedding of training content on topics such as multilingualism, German as a second language (DaZ), dealing with linguistic heterogeneity, and language-sensitive teaching. In some courses of study, corresponding modules are mandatory; in others they are rarely offered. In Baden-Württemberg, where the present study is located, student teachers can in some cases acquire an additional qualification for DaZ on an optional and voluntary basis (Fried and Gawlitzek, 2021). Even if teachers have acquired target group-specific professional knowledge during their teacher training, continuous in-service professional training is needed that connects to previous knowledge and practical experience (Abacioglu et al., 2022). If teachers do not have the target group-specific professional knowledge, additional training and thus additional effort is required.

Since preparation classes in Germany had to be set up from scratch for many refugees within a short time (Reinke and Goller, 2022), there was a great need for and lack of teachers (Jørgensen et al., 2021). For this reason, in addition to “regular” teachers, hirings were made also of language teachers, retired teachers, and people changing career. In this respect, a wide range of target group-specific professional knowledge, and of professional preparation for interacting with refugee students and for high-quality teaching can be assumed.

## The case of Baden-Württemberg in the German context

The data analyzed in this article were collected in one administrative district in the German federal state of Baden-Württemberg. In general, the regulations and institutional structure for the prevocational preparation classes differ between the individual federal states in Germany – from regulations of refugees' access to the educational system to the duration of schooling, the curriculum, and the possibility of obtaining a secondary school leaving certificate (Baethge and Seeber, 2016; Emmerich et al., 2017). These regulations also changed to some extent over time.

During the period that our data were collected, after young refugees arrived in an initial reception center for asylum applicants in Baden-Württemberg, they were subjected to an initial recording of their education biography. After a waiting period of up to 6 months, they were assigned to preparation classes. These classes exist for the general schooling of children in primary and secondary education, as well as for vocational schooling for youth and young adults. In Baden-Württemberg, all immigrant adolescents aged 16 to 20 (and in practice some even older), no matter whether they are refugees or other migrants, should attend these prevocational preparation classes called “Pre-qualification Year for Work and Occupation without German Language Skills” (VABO), at vocational schools. The prevocational preparation classes primarily aim to acquire language level A2 according to the Common European Framework of Reference for Languages (CEFR). In addition to language acquisition and literacy, teachers in these prevocational preparation classes were required to consider the psycho-social situation of their students and to facilitate their arrival in Germany. Refugee integration into the social life of their school as well as in the local community is fostered. After the prevocational preparation class, transition to a regular class is intended, for example to the regular education program “Pre-qualification Year for Work and Occupation” (VAB) in order to acquire the secondary school leaving certificate (MKJS, 2016, p. 12) to start vocational training or to attend an upper secondary school.

Even though the case of Baden-Württemberg may differ from other German federal states to some extent, all prevocational preparation classes in Germany pursue the overarching goal of German language acquisition, and provide an initial vocational orientation (Baethge and Seeber, 2016). Due to that and the fact of similar tasks and challenges (see Section The special role of teachers for the integration of refugees–Target group-specific professional knowledge of teachers) for teachers, it can be assumed that teachers in other parts of Germany share the experience of everyday working life to a certain extent, although the teachers interviewed here do not form a representative sample. This may also be true in an international perspective, since the education systems in all refugee receiving countries face the task of contributing to the integration of these refugees (see Section The special role of teachers for the integration of refugees). Nationally and internationally, the “unpreparedness” of educational systems and consequently of teachers to teach refugee students may also represent a common feature.

## Data and methods

A survey of teachers teaching in prevocational preparation classes for refugees at vocational schools investigated the integration of young refugees from a teachers' perspective. The survey was carried out in one administrative district in the German federal state of Baden-Württemberg, in the course of a student survey of the project “RISE (Refugees and their early Integration into Society and Education)”^5^ between May and September 2017. RISE investigates the early integration and educational pathways of refugees who are obliged to attend vocational school. For RISE, all vocational schools with prevocational preparation classes for refugees in one administrative district were invited to participate in the study in a two-step process. In total, 22 schools participated.

Teachers were invited to participate in the survey via a questionnaire during the on-site student survey by the survey team and after the survey by other teachers via snowball sampling. Participation was voluntary and could be undertaken on-site either at a computer or by paper-pencil. Subsequent participation of other colleagues was made possible by sharing links to the online questionnaire.

The semi-standardized questionnaire included, for example, questions about the teachers' education and professional background as well as questions about personal motivation for being a teacher in a prevocational preparation class. An assessment of the characteristics of the students in the prevocational preparation classes was also requested. This was followed by questions about stress, collegial support, and satisfaction with the job. Beside closed questions, which have already been established in other studies, three open questions were asked. In these questions, teachers could express themselves freely and in detail. The answers obtained provided in-depth insights into the pros and cons regarding their job in prevocational preparation classes as well as on important things that they have taught their students. The answers partly coincide with the closed questions but go beyond them and address additional aspects. With this approach, we combined the advantages of standardized, quantitative methods with the advantages of qualitative methods to provide a more comprehensive analysis of the situation for the teachers of refugees.

### Operationalization

Teachers' burdens were measured in two ways: First, teachers were asked to assess how much different dimensions of heterogeneity of the taught prevocational preparation class(es) stressed them on a 6-point scale (1 = not at all stressful to 6 = very stressful): “performance heterogeneity,” “linguistic heterogeneity,” “behavioral heterogeneity,” and “cultural heterogeneity composition”. In addition, the teachers were asked whether they felt they could cope with the stress they were exposed to in the prevocational preparation class. Likewise, a 6-point scale was used for this purpose (1 = not at all true to 6 = very true)^6^. Second, in an open question, they had the opportunity to express negative aspects of their job: “By which aspects of your job as a prevocational preparation class teacher are you bothered?”.

The gains that teachers experience when teaching in prevocational preparation classes were surveyed using an open question about positive aspects of their job: “Which aspects of your job as a prevocational preparation class teacher do you particularly like?”.

A combination of closed and open questions was also used to survey teachers' contributions to the integration of refugee students. The closed question captures the teachers' contributions rather indirectly via an assessment of the students' chances of success in the prevocational preparation class. This question acts as an indicator of the students' chances of integration in the four different integration dimensions according to Esser (2006): “learning the German language,” “learning specialized knowledge,” “obtaining a job or training position,” “making friends with Germans,” and “getting along in everyday life (e.g., with authorities)” (6-point scale: 1 = very low to 6 = very high). The open question, on the other hand, focuses more on the teachers' actions and asks about the most important aspects that the teachers have taught their students. The target group-specific professional knowledge was measured with a question about different professional training courses (multiple answers allowed): DaF (German as a foreign language) training; DaZ (German as a second language) training; professional training (interculturality, migration, trauma, etc.); language course; other. In addition, for the chosen training courses respondents were asked to provide information, whether the professional training took place before or during work as a teacher in a prevocational preparation class.

In the present study, in the absence of a scale to capture teachers' multicultural beliefs as a part of professional knowledge, teachers' attitudes toward immigration were used as an approximation. These were assessed with the question “Some people claim that immigration is more of a problem for Germany, others see it more as an opportunity for Germany. Which comes closest to your view?” (1 = immigration is more of a problem for Germany; 2 = immigration is more of an opportunity for Germany; 3 = immigration is equally an opportunity and a problem for Germany). This question is a well-established instrument that is used identically or in similar wording in different surveys on migration and integration (e.g., Transatlantic Trends: Immigration 2010, 2017 Special Eurobarometer).

### Sample and analytical strategy

Teachers from 60 prevocational preparation classes were invited to participate in the survey of which 48 teachers completed the questionnaire. Thirty-six respondents were female and 10 were male (two respondents did not indicate). On average, respondents were 46 years old at the time of the survey (SD = 13). About a quarter of the respondents (*n* = 13) were between the ages of 20 and 34. Slightly less than a quarter (*n* = 10) were in the 35 to 50 years age range, and just less than half of the teachers in prevocational preparation classes were between 51 and 65 years old at the time of the survey. Respondents had an average of 12 years of professional teaching experience (SD = 10). More than half of the respondents (*n* = 28) were classroom teachers in a prevocational preparation class at the time of the survey; 20 respondents taught in the prevocational preparation class on an hourly basis.

As a first step, we describe the completed target group-specific professional training by teachers, their attitudes toward immigration, their perception of stress and experienced burdens as well as their gains and contributions to their students' integration. In a second step, we examine group differences in the perceptions of teachers with and without target group-specific professional knowledge based on quantitative and qualitative results.

Therefore, the teachers' answers to the three open questions were coded and evaluated on the basis of a qualitative content analysis according to Mayring (2015) using the program f4analyse (version 2.5.6). We finally assigned the positive aspects of the job to six main categories, the negative aspects to seven main categories, and the taught content to eight main categories (plus subcategories in each case). To reflect the range of content, variation, and differentiation of the answers, we also determined for each teacher how many different categories his/her answers could be assigned to. This allowed not only a comparison between the teachers but also between their answers to the respective open questions.

We analyze the closed questions as well as the quantified categories from the open questions using descriptive statistics and, *t-*tests, or Mann-Whitney-U tests in the case of non-normally distributed variables. To account for the fact that, due to the sample size, effects may not turn out to be significant in small samples even though they are substantial and relevant to the content, the effect size was calculated in addition to the significance level. Effect size was calculated with Cohen's d for the *t-*tests where d ≥ 0.20 is a small effect, d ≥ 0.50 is a medium effect, and d ≥ 0.80 is a large effect (Cohen, 1988). For the Mann-Whitney-U tests, the effect size was determined based on the z-value and sample size using Pearson's correlation coefficient r. Thereby, r ≥ 0.10 corresponds to a small effect, r ≥ 0.30 to a medium effect, and r ≥ 0.50 to a large effect (Cohen, 1988). Analyses were performed using the programs Stata (version 14.2) and SPSS (version 28). A more detailed analysis of multivariate correlations could not be performed due to the small sample size.

## Results

### Teachers' professional knowledge

**Figure 1:**
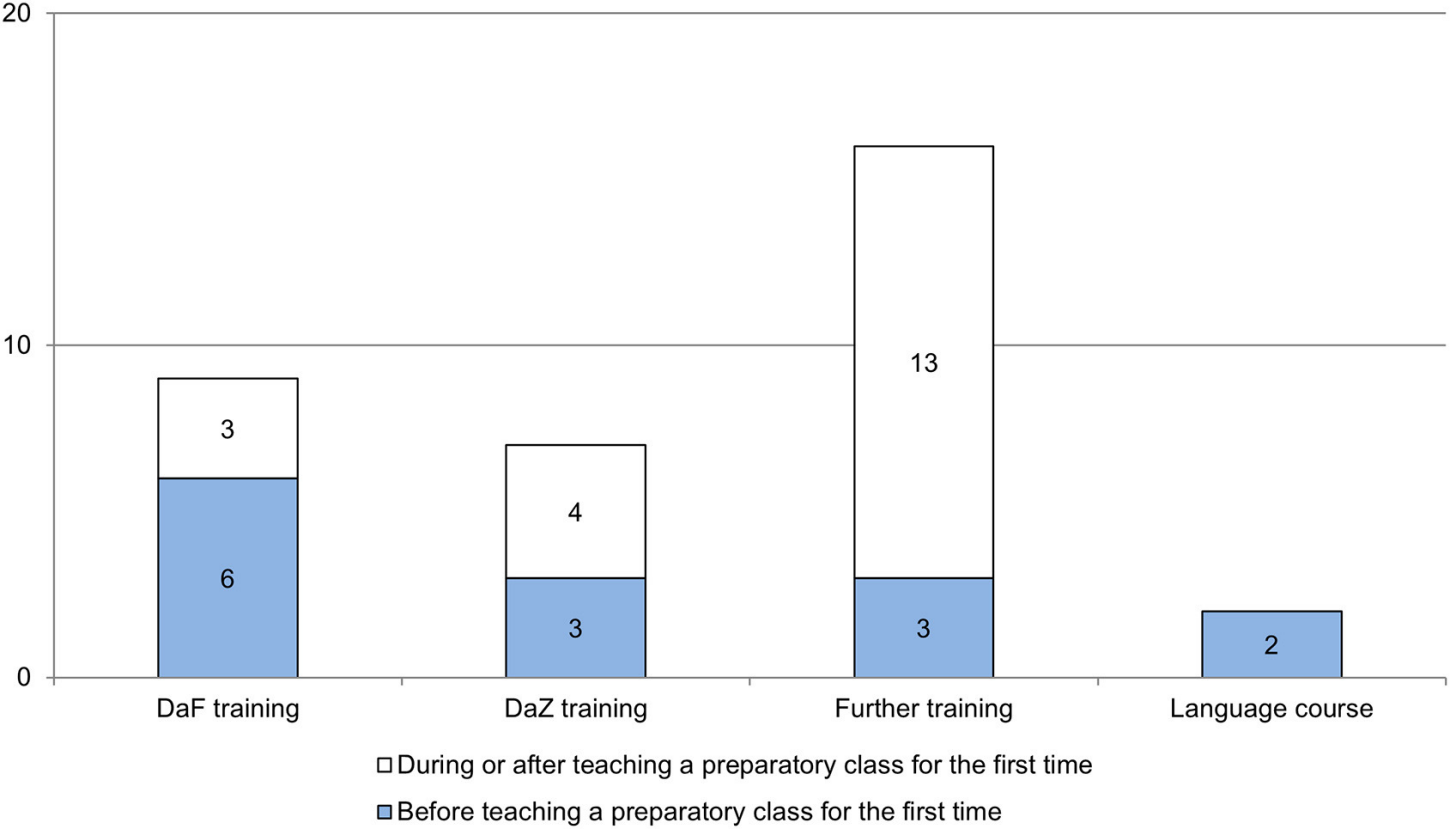
Target group-specific professional training according to time of completion (absolute frequencies; multiple answers possible; source: survey of teachers in preparation classes, *n* = 28).

Although target group-specific professional knowledge is essential to meet the particular requirements of teaching refugees (see Section Target group-specific professional knowledge of teachers), only 28 persons (58.3%) represented in the study have target group-specific professional knowledge as a result of professional training; 20 do not. The persons with target group-specific professional knowledge had already participated in 14 trainings before they started working in the preparation class for refugees. During or after the teaching of the first preparation class, another 20 trainings were completed (see Figure 1). Most teachers completed one training (71.4%); more than one in four completed more than two trainings (28.6%).

Most frequently, various professional training courses were completed (*n* = 16). These courses might have included, for instance, training on dealing with traumatized students or culturally heterogeneous classes. Teachers also took DaF (*n* = 9) and DaZ training courses (*n* = 7) – courses to teach German as a foreign or second language. Very few teachers had attended a language course (*n* = 2). DaF training and language courses tended to be completed before starting to work in the preparation class, while topic-specific professional training was mainly attended during or after teaching a preparation class for the first time. The open answers concerning teachers' burdens in preparation classes for refugees reveal that teachers' own education or the education of their colleagues is an important topic for them (see Section Teachers' burdens). For example, teachers criticized colleagues who in their opinion did not possess the necessary education.

**Figure 2:**
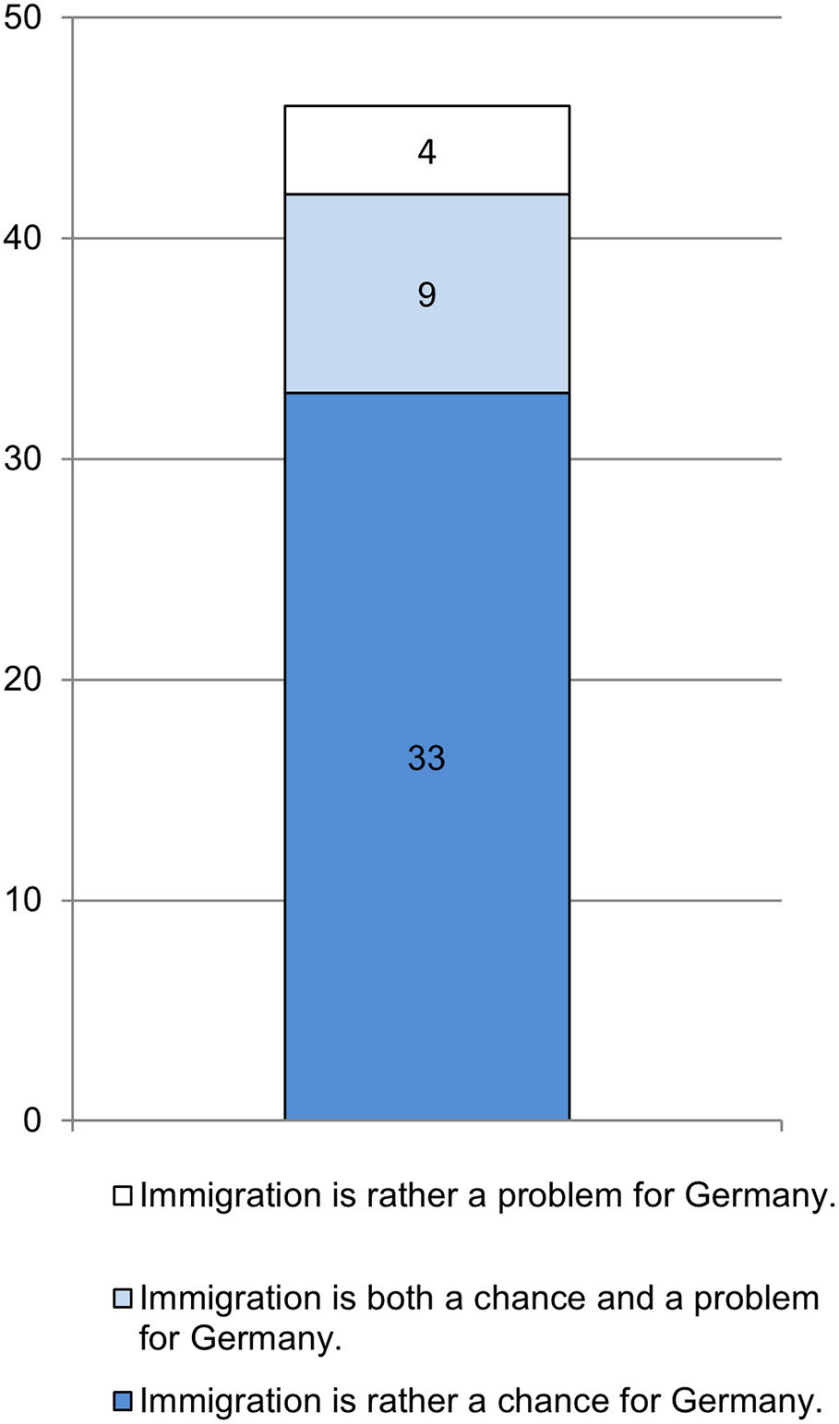
Immigration as an opportunity or a problem? (absolute frequencies; source: survey of teachers in preparation classes, *n* = 46).

With regard to attitudes as part of teachers' professional competence, it appears that nearly three quarters of the teachers surveyed (71.1%) rated immigration as both an opportunity and a problem for Germany (see Figure 2). Only four teachers (8.7%) saw immigration exclusively as a problem while the remaining nine saw it solely as an opportunity (19.6%). In the open answers on gains, teachers emphasized how their instruction and their own personality benefited from the cultural diversity in the classroom and intercultural exchange.

The view of teachers regarding both opportunities and barriers to immigration is also reflected in the reported positive aspects (see Section Gains in teaching refugees) and specific burdens of their job (see Section Teachers' burdens) as well as the anticipated integration chances of their students (see Section Teachers' contributions to students' progress and integration).

### Teachers' burdens

The open question on negative aspects of the job was answered by 41 teachers (85.4%). The responses range from two teachers stating that nothing concerned them at the moment to two teachers listing each six different negative aspects about their job. On average, the teachers' answers can be assigned to M = 2.49 (SD = 1.45, *n* = 41) categories. Thus, on average, teachers mention one category less in the negative aspects than in the positive aspects of their job.

Most of the comments refer to clear criticism of the working conditions (*n* = 37), such as fixed-term contracts with short terms and no employment during the summer vacations.

*We are only a means to an end. As long as we are needed, we are hired and then only for a limited period of time; during the summer vacations we are dismissed. This is an impertinence. We do just as much, if not more, work than other teachers. (teacher with DaF training and target group-specific professional training)*

Furthermore, some teachers felt that their work was not valued and that they were not supported in their role, whether by colleagues, superiors, or the authorities. One teacher's comment that “over-committed volunteers” interfere in her work also points to the lack of recognition of her own expertise and a corresponding disregard for the work performed on the part of third parties. In addition, the use of colleagues from other disciplines without the expertise and motivation necessary for teaching this specific target group was criticized, or the lack of their own expertise was indicated.

Further criticisms referred to a view of the students as deficient and the pressures exerted by politics—both on the students and on the teachers.

*Pressure from politics—students have to learn quickly because of papers etc./no flexible handling (age/learning level/ability to learn –*> *everyone has a right to education?!) (teacher with DaF training and target group-specific professional training)*

This corresponds to the comments that regulations from authorities, agencies, and ministries demand “a high degree of flexibility” from teachers (teacher with target group-specific professional training). Criticism of unclear, inconsistent, and changing specifications for preparation classes all point in the same direction. On the one hand, the guidelines are not sufficiently well communicated (*n* = 15), and on the other hand the work required of the teachers does not comply with the reality at school in preparation classes and the needs of the students.

*The teaching hours for the preparation classes have been cut. Unfortunately, there are no physical education classes and no practical subjects. Six h of German lessons in one school day is too much for some students. (teacher with target group-specific professional training)*

The lack of suitable teaching materials^7^ as well as too much time spent on administration, organization, and social work, make the role even more difficult.

*Partially inadequate teaching materials (textbooks sometimes plucked out of thin air and therefore often inconclusive) (teacher with DaZ and DaF training)*

In addition, insufficient training or professional training of the teachers (*n* = 5) is noted, both among the teaching staff and the interviewed teachers (see above).

The dissatisfaction with administrative regulations is reflected in statements about the classes (*n* = 14) which primarily refer to regulations regarding the composition and excessive heterogeneity of the students, for instance with regard to performance, willingness to perform, and educational background. Dealing with the heterogeneous students as well as with disturbances to a calm working atmosphere is challenge for the teachers.

*I can never work with a fixed class community because the students are somehow “transient”. They come, they move, they get deported.... This creates disturbance and not a good learning environment. (teacher with target group-specific professional training)*

With reference to students (*n* = 26), the main issues mentioned are lack of motivation, difficulties with discipline, high absenteeism, and lack of secondary virtues (e.g., punctuality), as well as difficulties regarding learning and performance.

*In each of our classes there are students (not female students) who are often absent, show no interest, behave arrogantly and condescendingly, and do not follow any rules. They ruin a lot as a result. (teacher without target group-specific professional training)*

In addition, there is trauma, a lack of continuity due to residence regulations and deportations, and a lack of contact with German students. All of this can have a negative impact on learning progress (*n* = 3) and the relationship between teachers and students (*n* = 5).

*The “struggle” with the many absences and tardiness as well as the respect that has to be gained, especially at the beginning and actually always. (teacher with DaF training)*

**Figure 3:**
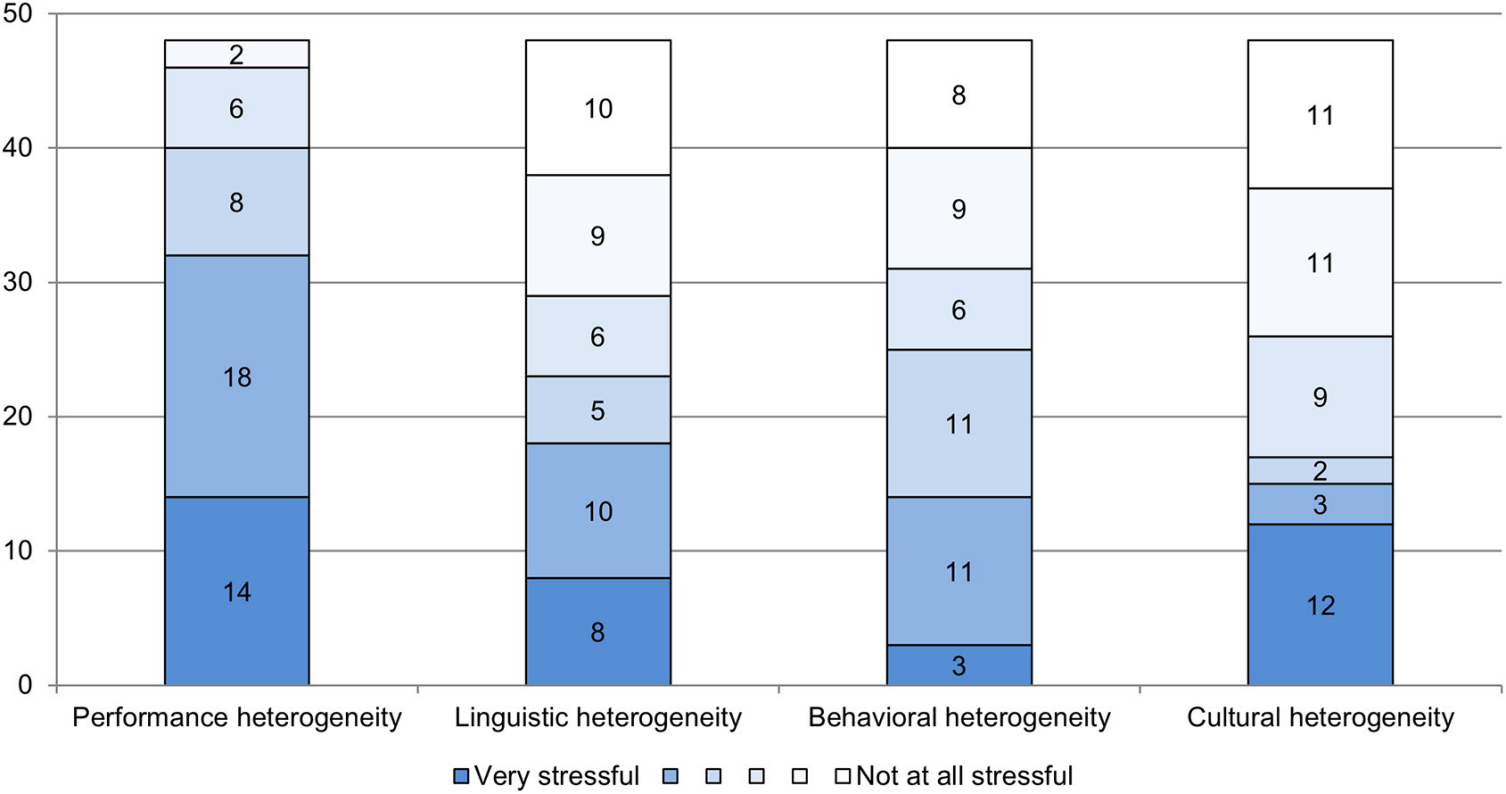
Perception of stress with regard to various aspects of class heterogeneity (absolute frequencies; scale from 1 = not at all stressful to 6 = very stressful; source: survey of teachers in preparation classes, *n* = 48).

The responses to the open question about perceived challenges of teaching in prevocational preparation classes that relate to the students and the class are consistent with the standardized assessed challenges due to class composition. Teachers perceived the performance heterogeneity of classes as by far the greatest burden (M = 4.75, SD = 1.14, *n* = 48) (see Figure 3). Slightly more than half of the respondents perceived heterogeneity in terms of behavior (M = 3.35, SD = 1.58, *n* = 48) as rather burdensome. The situation is similar with linguistic heterogeneity (M = 3.42, SD = 1.83, *n* = 48) which is perceived as rather burdensome by just under half the respondents. Cultural heterogeneity of the students caused the least sense of stress (M = 3.23, SD = 1.93, *n* = 48). In general, few teachers felt they could not handle the stress they face as a teacher in a preparation class. The vast majority of teachers rated their stress resilience as high (M = 5.19, SD = 0.17, *n* = 47).

The comparison of teachers with and without target group-specific professional knowledge showed no significant differences regarding the stress caused by behavioral heterogeneity (t(46) = −1.52, *p* = 0.14, *n* = 48, d = −0.45) and by cultural heterogeneity (z = −1.27, *p* = 0.20, *n* = 48, r = −0.18), and the effect sizes were small. Teachers with target group-specific professional knowledge perceived greater stress with regard to performance heterogeneity (z = −1.97, *p* = 0.05, *n* = 48, r=-0.28) and perceived greater stress with regard to linguistic heterogeneity (t(46) = −1.86, *p* = 0.07, *n* = 48, d = −0.54) than teachers without such knowledge and training; in the first case the effect size was small, in the second case the effect size was medium. Regarding the feeling of being able to cope with stress as a whole, the two groups did not differ significantly nor was the associated effect size substantial (z = −0.50, *p* = 0.62, *n* = 45, r = −0.07).

In contrast to the answers to the closed questions, the answers to the open question were not statistically significantly different between the two groups of teachers (z = −1.13, *p* = 0.28, *n* = 41, r = −0.18). Compared to teachers with target group-specific professional knowledge (M = 2.33, SD = 1.44, *n* = 27), the responses of teachers without target group-specific professional knowledge were greater in number (M = 2.79, SD = 1.48, *n* = 14).

### Teachers' gains

The burdens of teaching in preparation classes for refugees (see Section Teachers' burdens) are contrasted with numerous positive aspects of the job. Almost all teachers (*n* = 41; 85.4%) answered the open question about positive aspects of their job. With regard to the personality of the students (*n* = 25), their motivation to learn, joy of learning, willingness to integrate, and friendliness are emphasized. In addition, tasks related to their phase of adolescence play a role.

*The students are very friendly, willing, and disciplined. (teacher with target group-specific professional training)*

The relationship between teacher and students (*n* = 31) is described by the former as trusting, personal, and characterized by the gratitude of the students. A good relationship between teachers and students is the basis for some refugees to ask their teachers for advice and support in extracurricular matters.

*I think it's nice to meet people from Asia and Africa who have given up a lot to start over. I have great respect for the life achievements of these 17–20 year-olds. Many of them have experienced terrible things and yet are very polite and thank you after every lesson. (teacher without target group-specific professional training)*

Statements about the class (*n* = 16) refer primarily to a constructive working atmosphere and an appreciation of cultural diversity through which the teachers (*n* = 27) themselves learn a lot and develop personally. They experience their work as a meaningful task and themselves as supporters of integration processes.

*The linguistic and cultural diversity that the students bring with them, and this leads to lively exchange. In this way, not only do the students learn about Germany but I, too, can learn new things or reflect on familiar ones and encourage the students to do the same. (teacher with target group-specific professional training)*

*The intensive and social cooperation with students. I get to know the students (their personality, background) intensively and can encourage and challenge them. I am glad that I am allowed and able to do this work, because this way I can help them in different areas/skills. (teacher with target group-specific professional training)*

The teachers see progress in the students (*n* = 22) with regard to various aspects of integration as well as, for example, learning or knowledge acquisition.

*Important contribution to integration / profitable interaction/observable progress* –> *language skills, subject knowledge and social behavior (teacher without target group-specific professional training)*

What the teachers (*n* = 28) value most about their work is the freedom they have in terms of the content and methods when designing lessons. On the one hand, the fact that they do not have to adhere to prescribed curricula and do not have to do time-consuming corrections of class work allows them to respond individually to the different needs of the students. On the other hand, it offers them variety.

*Freedom to design lessons themselves and to set priorities, to be able to react spontaneously to situations in the classroom (teacher with DaF training)*

The teachers emphasize the high value of “pedagogical work,” in part also to distinguish the lessons from regular classes. The pedagogical work is related to a good relationship between teacher and students. Two teachers point to collegial cooperation or team teaching which is experienced as profitable and successful. Teachers get various gains from their job of teaching refugees. More than one third of the teachers named only one or two aspects, while some others named more, and one person selected eight categories. On average, the teachers' answers could be assigned to M = 3.49 (SD = 1.87, *n* = 41) categories. Similar to the question about negative aspects of the job, teachers without target group-specific professional knowledge answered in a more differentiated way. They named more aspects that they liked about their job (M = 3.81, SD = 2.01, *n* = 16) than did teachers with target group-specific professional knowledge (M = 3.28, SD = 1.79, *n* = 25). However, the difference between both groups was small and not statistically significant (z = −0.89, *p* = 0.39, *n* = 41, r = −0.14).

### Teachers' contributions to students' progress and integration

Despite the many challenges, 43 teachers (89.6%) also mentioned important things that they taught their refugee students. More than half of the teachers indicated only one or two aspects, just under one-third indicated three or four aspects, and the remainder indicated five and eight aspects. On average, the teachers named just under three aspects (M = 2.84, SD = 1.81, *n* = 43). With regard to the students' personalities (*n* = 36),^8^ they observed a strengthening of self-confidence and independence, more competent speaking about problems and solving problems, increased resilience and more joie de vivre. Several teachers attest to their students' willingness to integrate. With regard to educational processes, teachers report an increase in concentration span and the development of a positive attitude toward learning. Teachers attribute great importance to the acquisition of secondary virtues (e.g., discipline and punctuality) and a willingness to make an effort. Furthermore, the teaching of tolerance and respect is emphasized.

*Respect & tolerance for all religions & ways of life (including homosexuality), recognizing and taking the opportunity of freedom here in Germany as far as possible (teacher with target group-specific professional training)*

This is closely related to the teaching of values and rules (*n* = 8). For the teachers, it is essential that the young refugees accept basic rules and values of living together in Germany, such as the equality of men and women or the role of religion.

*I have to know the rules in Germany. The Basic Law lays down these rules. Even if women's emancipation, homosexuality, and Israel's right to exist are foreign to me, I have to understand that these aspects are part of German culture. (teacher without target group-specific professional training)*

There are clear overlaps between the teachers' answers in the category of values and rules and aspects of intercultural sensitization and competence (*n* = 14). On the one hand, these refer to the teaching of “German habits” and to finding one's way in German culture. On the other hand, comparisons are made between Germany and the countries of origin to classify situations and recognize any misunderstandings or problems that may arise.

*That many things that have always been considered absolute truths are subject to cultural imprinting and are very individual. Religion, role models of men and women, ideas of obedience and respect have to be discussed. Everyone has learned to at least listen and put their own truths into perspective. (teacher without target group-specific professional training)*

In addition to helping students find their way in a new country by developing their personalities, teaching them tolerance, values, and rules, and raising their intercultural awareness, teachers also consider it important to help their students cope with everyday life (*n* = 5).

*Survival training (knowledge of essential things needed to get along in everyday life/with authorities as well as in dealing with Germans, that you talk about your problems when the time is right and that you can look for and find a solution together (teacher with DaZ training)*

The special importance of language becomes clear in 33 statements that refer to language in general or to individual dimensions (speaking, reading, writing, understanding). There are also references to the importance of language for communication and understanding as a prerequisite “for everything else”.

*Reading* + *writing as key competencies for our educational society (teacher without target group-specific professional training)*

Teachers who teach literacy classes face the difficult task of having to teach the cultural techniques of reading and writing in addition to the German language.

*I teach illiterate people. Now, after one school year, they can read and understand simple texts and answer questions about them. They can also write simple sentences. In addition, they can communicate quite well orally. (teacher without target group-specific professional training)*

Closely related to statements about language skills are statements about contacts (*n* = 13). These are not aimed exclusively at contacts with Germans, but also at interaction with one another and social competence.

*Language competence and performance; to dare to start a conversation, to lead a conversation (teacher with target group-specific professional training)*

*That they are able to communicate in the simplest of sentences and that they have become accustomed to the structure of a class and to the structures of a school (teacher without target group-specific professional training)*

In addition, the structures in school and in lessons as well as the behavior of the teachers (*n* = 9) provide the necessary framework both for arriving in a foreign country and learning processes.

*That school is a reliable constant that can help to cope with everyday life and understand German culture in addition to learning the language (teacher with target group-specific professional training)*

With regard to successful learning processes (*n* = 9), subject-related aspects such as the acquisition of the German language, or knowledge of mathematics and supra-subject-related aspects, such as learning to learn and feeling joy in this process, are stated.

*For me, it's more about working together. There are many different nations in the classroom. Mutual consideration and complementing others regarding their learning disadvantages (language, skills...) and coming together to achieve the teaching goal. (teacher with DaZ training)*

**Figure 4:**
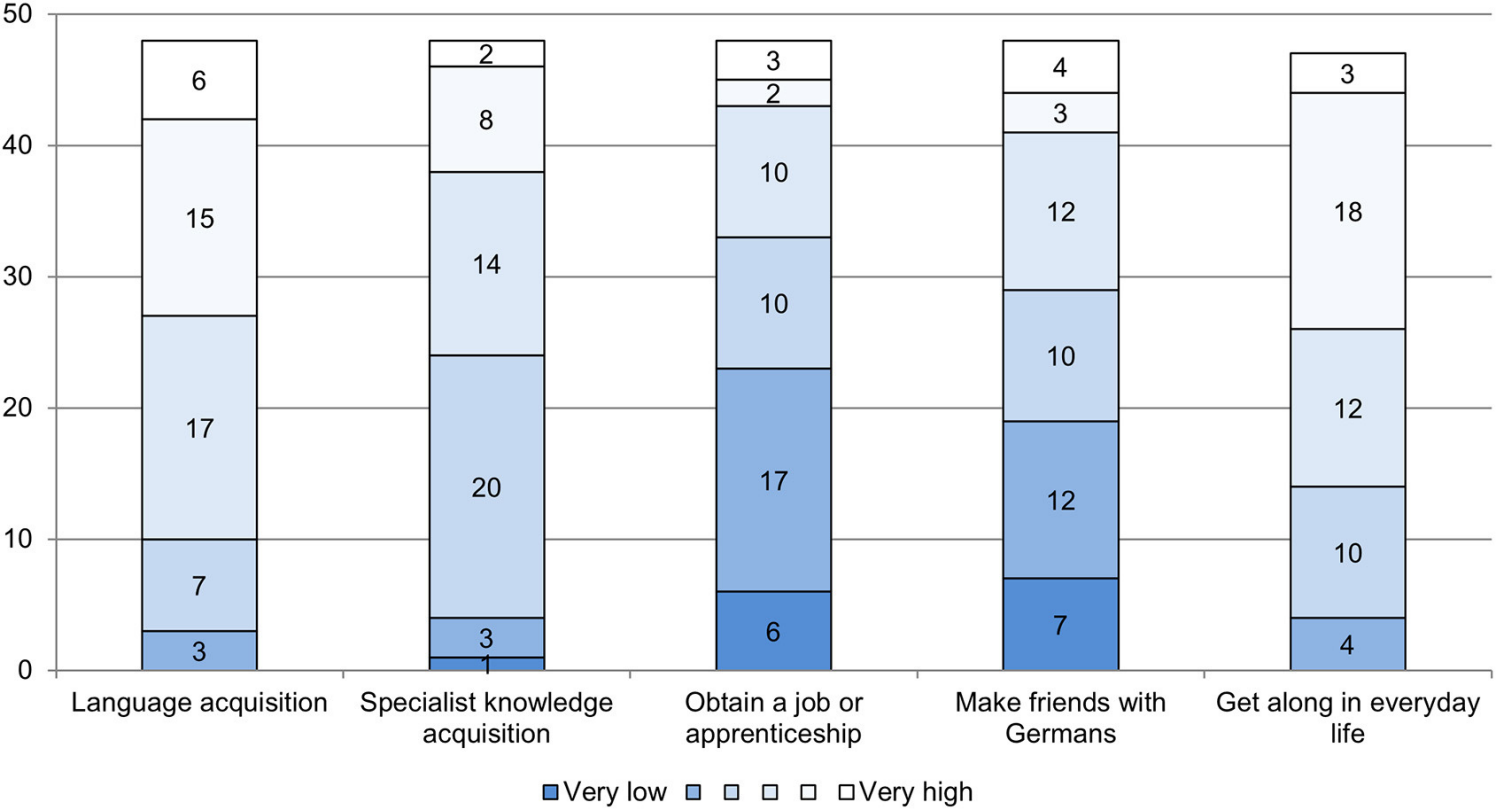
Assessment of students‘ chances of success in integration (absolute frequencies; scale from 1 = very low to 6 = very high; source: survey of teachers in preparation classes, *n* = 48).

In line with the special importance of language for integration (Esser, 2006) and their educational mission regarding language acquisition, most teachers emphasized in this open question that they had been able to teach their students something in this regard. First, this finding fits with the fact that when asked what they value about their job, teachers respond to their students' progress in learning, language acquisition, and integration (see Section Gains in teaching refugees). Second, this result is also reflected in the standardized assessment of their students' chances of success as an indicator of their own teaching skills and as a contribution to integration in a broader sense. The teachers saw the highest chances of success for the refugee students by acquiring the German language (M = 4.29, SD = 1.07, *n* = 48) and in getting along in everyday life (M = 4.13, SD = 1.10, *n* = 47) (see Figure 4). The chances of success in acquiring specialist knowledge (M = 3.65, SD = 1.04, *n* = 48) were rated at least as rather high by half of the respondents; the other half was more skeptical in this respect. The chances of making friends with Germans were rated lower (M = 3.08, SD = 1.46, *n* = 48) and the chances of obtaining a job or an apprenticeship were rated the lowest (M = 2.88, SD = 1.35, *n* = 48).

Looking at the relationship between target group-specific professional knowledge and perception of students' chances, there were no significant differences between teachers with and without professional training. Teachers without target group-specific professional knowledge tended to assess their students' chances of success in acquiring the German language better than teachers with target group-specific professional knowledge. This difference was not significant, and the effect size was small (t(46) = 0.86, *p* = 0.39, *n* = 48, d = 0.25). Likewise, the estimated chances of success in acquiring specialized knowledge did not differ significantly between the two groups (t(46) = 0.02, *p* = 0.98, *n* = 48, d = 0.01). Referring to labor market success (t(46) = −0.76, *p* = 0.45, *n* = 48, d = −0.22), making friends (t(46) = −0.53, *p* = 0.60, *n* = 48, d = −0.16), and getting along in everyday life (t(45) = −0.41, *p* = 0.68, *n* = 47, d = −0.12), the chances of success of refugee students were assessed as comparatively better by teachers with target group-specific professional knowledge. However, even here the differences between the two groups were not statistically significant. In fact, the only small effect is in the case of labor market success.

Qualitative analyses of the open responses to the question about teaching success also revealed non statistically significant and only small differences between the two groups (z = −1.36, *p* = 0.17, *n* = 43, r = −0.21). In contrast to the two open questions about the positive and negative aspects of the profession, teachers with target group-specific professional knowledge reported more important things that they were able to teach their students (M = 3.08, SD = 1.74, *n* = 26) than did teachers without target group-specific professional knowledge (M = 2.47, SD = 1.91, *n* = 17).

## Discussion

Structural integration into (vocational) education and the labor market (Esser, 2006) is not only crucial for independent living but at the same time an important task of adolescents (e.g., Stöbe-Blossey et al., 2019). “Participation—more precisely: occupational and social participation—thus becomes the central target and content category of education” (Werner, 2017, p. 7). Young refugees do not only have to get economically independent as does almost every young adult, they also have to make their way through the education system and into the labor market of a country that is new to them in terms of, for example, the language spoken and the cultural norms. Our article focuses on how teachers of refugee youths support their students in reaching these milestones and how the teachers experience their work. Teachers have a special role as representatives of the majority society, as important contact persons for the students and as those who shape the educational and integration context (e.g., Blossfeld et al., 2016; Sari and Kenner, 2017). First, this article contributes to the description of the burdens, gains, and contributions to their refugee students' integration of teachers who teach in special preparation classes at vocational schools. Second, it sheds light on the teachers' target group-specific professional knowledge and on the extent to which this knowledge shapes the teachers' perceived burdens and gains in teaching young refugees as well as their students' integration chances. Third, by combining results from qualitative and quantitative analyses, we can depict teachers' gains and burdens as well as pointing to structural and institutional weaknesses.

Our study shows that teachers of refugees, the stress and burdens of their job notwithstanding, also gain positive experiences during their work. For example, they take pleasure in the lack of guidelines and resources, enjoying their freedom in choosing teaching contents and methods. This finding and the appreciation of the students' motivation and gratitude conforms to the findings of Simml (2019) in Bavarian prevocational preparation classes for refugees. In the open answers, teachers also emphasize positively that they observe considerable progress in learning and in the integration of their students. Especially as regards the acquisition of the German language, which Esser (2006), p. 52; describes as a “central condition for any further social integration” (translation by the authors), and the coping with everyday life, the teachers consider the students' chances of success to be high in the closed questions. At the same time, in the open responses, the teachers mentioned these aspects as areas in which they were able to teach their students important knowledge. This is possibly related to the fact that, according to the guidelines for the preparation class (MKJS, 2016), teachers are responsible for exactly these areas, feel responsible for them, and spend a corresponding amount of class time on them. In addition, the open statements suggest that some teachers have a trusting relationship with the students (Sari and Kenner, 2017; Simml, 2019; similar to Reinke and Goller, 2022), such that they also invest time in counseling and guidance outside the class and support integration processes (Karakayali et al., 2017). Here, the answers provided by the teachers on these topics might be confounded to some extent by an assessment of their own performance or contributions to the success of the students.

The assessments of success in different areas—higher chances of success in culturation, lower in terms of placement and interaction (Esser, 2006)—show the limited scope of the teachers' actions: while they can stimulate the acquisition of the German language in their lessons and convey the value of secondary virtues, their ability to support the students, e.g., with obtaining apprenticeships or initiating contacts with natives, are significantly lower. However, with regard to social integration, Grütter et al. (2021) show that teachers of fourth graders at German elementary schools are key to establishing contacts between students of different ethnic groups. Our findings may indicate an underestimation of the influence of teachers in this regard. Another explanation could lie in differences between schools: While Grütter et al. (2021) studied ethnically heterogeneous classes including native children, the schooling of refugees in the case of our study took place separately without institutionalized contact with native students. Regardless of this, teachers and students are dependent on the openness of the society toward refugees—an openness that is prevalent among the German population (SVR, 2018).

Another prerequisite for the integration of refugees, from the perspective that integration is a two-way process, is the provision of resources on the part of the host society. For the schooling of refugees examined here, this means, among other things, not only that a sufficient number of teachers is employed under good working conditions, but also that these teachers have the necessary qualifications and target group-specific professional knowledge. This is the only way to realize the guiding principle of simultaneous support and challenge for the students. The comments of the teachers, however, show that the practical implementation looks different. First, as the numerous criticisms of the framework conditions for teaching refugees mentioned in the question about negative aspects of their job make clear, teachers in prevocational preparation classes work under particularly challenging conditions (also Reinke and Goller, 2022). Second, only a little more than half of the teachers surveyed had completed professional training tailored to the specific target group, and thus enhanced their migration-specific knowledge as well as their pedagogical-psychological competence (Voss and Kunter, 2013). This is consistent with findings by Karakayali et al. (2017) on teachers in refugee classes at Berlin elementary schools in which the majority were lateral entrants who also had a wide range of prior experience and prior knowledge or specific training. On the one hand, this shows that teachers with the relevant knowledge were specifically assigned to the preparation classes. On the other hand, the findings indicate that the majority of professional training was taken during the teaching of the first preparation class. This may result from an increase in opportunity structures due to the activity in the preparation class as well as a growing need for knowledge and skills that go beyond the teaching of language (MKJS, 2016; Karakayali et al., 2017). Additionally, a broad(er) range of appropriate professional training may only have been developed and provided with sufficient capacities as a result of an emerging need, so that some teachers could only participate with a certain delay.

However, the findings also point to the fact that a considerable share of teachers take on this challenging task without prior target group-specific professional knowledge, which is in line with previous findings (Reinke and Goller, 2022). This may be due to the fact that there were not enough teachers with target group-specific professional knowledge available for the large number of classes needed within a very short time. The open answers highlight that both insufficient own target group-specific professional knowledge and lack of target group-specific professional knowledge of colleagues lead to difficulties (Damaschke-Deitrick et al., 2022). This underlines the importance of well-founded training of all teachers on relevant topics, such as language-sensitive teaching (e.g., Gibbons, 2015; Becker-Mrotzek et al., 2021). With regard to the challenges the teachers experience, their high perception of stress as a result of performance heterogeneity in the students may come as little surprise given that the refugees came from different countries with different school systems and school attendance options. This finding follows previous reports that deal with different dimensions of heterogeneity in preparation classes for refugees is a challenge for teachers (Kärner et al., 2016). Teachers in the present study also mentioned in their open responses challenges in dealing with heterogeneous students. Though, criticism of working conditions, similar to Karakayali et al. (2017), and of students' behavior such as their lack of discipline and punctuality (Reinke and Goller, 2022), are significantly more in focus. This points to the fact that additional aspects of class composition, such as high turnover in classes (Scheiermann and Walter, 2016; Karakayali et al., 2017; Asghari, 2022), are also relevant and should be given more attention. On the macro level, the results can also confirm critical voices from the literature that regard preparation classes for refugees as a low-cost solution not necessarily meeting the prerequisites and needs of the students (Glorius and Schondelmayer, 2019, p. 225). However, to some extent the latter also applies to the teachers given that they have to fulfill their pedagogical task under difficult conditions.

The quantitative finding that the burden of heterogeneity in students' performance is perceived stronger among teachers with target group-specific professional training than among teachers without could be due to a greater awareness of the problem. Greater target group-specific professional knowledge and the associated opportunities for action resulting from their professional training could also play a role (cf. Vigren et al., 2022). The perceived low stress caused by the cultural heterogeneity of the students can be attributed to professional training, for example on intercultural competence, or to a generally more open attitude of the teachers toward immigration and immigrants (cf. Civitillo et al., 2019; Gebauer and McElvany, 2020; Hachfeld and Syring, 2020). The latter is also true of the teachers interviewed here, hardly any of whom saw immigration exclusively as a problem. The appreciative answers of the teachers in the open questions regarding cultural diversity in the preparation classes and the resulting individual learning and reflection processes attest to this. Regardless of whether or not the teachers have completed at least one piece of target group-specific professional training, they feel generally up to the challenges associated with teaching refugee students. Also, both groups do not differ when it comes to gains and burdens of their job: both mention more positive than negative aspects.

Whether teachers teaching in preparation classes differ from teachers teaching (exclusively) in regular classes, for example in terms of their multicultural beliefs, is presently unknown. Equally unclear is the transferability of our findings to teaching in preparation classes in other school types and other teaching levels, as well as associated differences of the students and schooling concepts (e.g., integrated instruction). In general, there is no Germany-wide representative study of teachers in preparation classes for refugees, so that we have to fall back on the findings from regional studies. However, since our findings are in line with findings in other German federal states for vocational schools (Simml, 2019; Reinke and Goller, 2022) as well as for elementary schools (Karakayali et al., 2017), they can be combined to form a bigger picture. This picture is also consistent with findings from other countries, where teachers teach refugees under often very different conditions (e.g., Khansa and Bahous, 2021 for Lebanon; Asghari, 2022 for Sweden). All studies share the finding that teachers in preparation classes enjoy teaching refugee students and are successful with their students, even if the less than optimal organizational conditions could be improved. In this respect and given the small sample size, the present article should initially be regarded as an exploratory starting point for further studies. Such studies should use both quantitative and qualitative methods to survey more comprehensively and further explore aspects addressed here.

Nevertheless, the current article provides initial insights into teaching preparation classes for refugees and highlights the importance of school attendance for the integration of refugees as well as the special role of the teachers to support this objective. Particularly, it points out the crucial relevance of teacher training (Lipowsky, 2011) in the context of educating refugees. By completing further training, teachers increase their professional knowledge and thereby raise their awareness of refugees' problems so that they can meet the key challenges of their job successfully. Further, the article stresses factors that negatively affect the wellbeing of teachers in preparation classes for refugees and therefore may have detrimental impact on their teaching performance and thus on their students' educational achievements. There is scope for action by policymakers to reduce at least some of the mentioned stressors by, as far as possible, equalizing the circumstances of teachers in preparation classes with those of regular teachers in Germany. This concerns salary and, more general, occupational security as well as creating a supportive institutional setting that provides course materials and clear, binding curricula. In view of the current desperate migration by countless mothers and their children triggered by the war in Ukraine, the challenges for all involved are renewed. At least the integration of these children into the education system can now build on past experience.

## Data availability statement

The raw data supporting the conclusions of this article will be made available by the authors upon request, without undue reservation.

## Author contributions

All authors listed have made a substantial, direct, and intellectual contribution to the work and approved it for publication.

## Conflict of interest

The authors declare that the research was conducted in the absence of any commercial or financial relationships that could be construed as a potential conflict of interest.

## Publisher's note

All claims expressed in this article are solely those of the authors and do not necessarily represent those of their affiliated organizations, or those of the publisher, the editors and the reviewers. Any product that may be evaluated in this article, or claim that may be made by its manufacturer, is not guaranteed or endorsed by the publisher.

## Footnotes

^1^The term “professional training” is understood to include both continuing and advanced education as well as specific training. The term “target group-specific professional training” encompasses various further education and training courses on migration and refugee-specific topics, such as German as a second language, intercultural training, or trauma in students (see also Section Target group-specific professional knowledge of teachers and Operationalization).

^2^In purely formal terms, vocational training is also possible without a school leaving certificate (Schier, 2017, p. 2).

^3^Internal differentiation (or internally differentiated instruction) refers to measures that adapt instructional content and learning arrangements to the various needs of students in a class. This can be done, for example, by means of differentiation through learning plans, grouping, and didactics (tasks, methods, etc.) (Paradies and Linser, 2019).

^4^As an example, we refer to the online course “Trauma in the Context of Flight and Asylum – Challenges in Non-Therapeutic Professions” of Ulm University Hospital and the University of Bielefeld, which is recognized as a continuing education course for teachers (https://shelter-trauma.elearning-kinderschutz.de; accessed 07/16/2021).

^5^More information on the project RISE can be found at https://www.rise-study.com.

^6^The questions, answer categories, and open answers from the German questionnaire were translated by the authors. In three cases, minimal changes were made to quotations in order to increase comprehension (e.g., “D” as an abbreviation for Germany was replaced by “Germany”).

^7^Further quantitative analyses reveal that 44 teachers (91.7%) created materials for teaching in preparation classes. 27 teachers (57.5%) received teaching materials from their school, 20 teachers (42.6%) were not provided materials by their school.

^8^The data refer to the number of responses that could be assigned to this category.
